# Genetics and Epigenetics of Parathyroid Carcinoma

**DOI:** 10.3389/fendo.2022.834362

**Published:** 2022-02-24

**Authors:** Francesca Marini, Francesca Giusti, Gaia Palmini, Giuliano Perigli, Roberto Santoro, Maria Luisa Brandi

**Affiliations:** ^1^ Department of Experimental and Clinical Biomedical Sciences, University of Florence, Florence, Italy; ^2^ Fondazione Italiana per la Ricerca sulle Malattie dell'Osso (F.I.R.M.O.) Italian Foundation for the Research on Bone Diseases, Florence, Italy; ^3^ Department of Experimental and Clinical Medicine, University of Florence, Azienda Ospedaliero-Universitaria (AOU)-Careggi, Florence, Italy

**Keywords:** parathyroid carcinoma, gene mutation, tumor suppressor genes, molecular signatures, epigenetic changes, DNA methylation, microRNAs, long non-coding RNAs

## Abstract

Parathyroid carcinoma (PC) is an extremely rare malignancy, accounting less than 1% of all parathyroid neoplasms, and an uncommon cause of primary hyperparathyroidism (PHPT), characterized by an excessive secretion of parathyroid hormone (PTH) and severe hypercalcemia. As opposed to parathyroid hyperplasia and adenomas, PC is associated with a poor prognosis, due to a commonly unmanageable hypercalcemia, which accounts for death in the majority of cases, and an overall survival rate of 78-85% and 49-70% at 5 and 10 years after diagnosis, respectively. No definitively effective therapies for PC are currently available. The mainly employed treatment for PC is the surgical removal of tumoral gland(s). Post-surgical persistent or recurrent disease manifest in about 50% of patients. The comprehension of genetic and epigenetic bases and molecular pathways that characterize parathyroid carcinogenesis is important to distinguish malignant PCs from benign adenomas, and to identify specific targets for novel therapies. Germline heterozygote inactivating mutations of the *CDC73* tumor suppressor gene, with somatic loss of heterozygosity at 1q31.2 locus, account for about 50-75% of familial cases; over 75% of sporadic PCs harbor biallelic somatic inactivation/loss of *CDC73*. Recurrent mutations of the *PRUNE2* gene, a recurrent mutation in the *ADCK1* gene, genetic amplification of the *CCND1* gene, alterations of the PI3K/AKT/mTOR signaling pathway, and modifications of microRNA expression profile and gene promoter methylation pattern have all been detected in PC. Here, we review the current knowledge on gene mutations and epigenetic changes that have been associated with the development of PC, in both familial and sporadic forms of this malignancy.

## Parathyroid Carcinoma

Parathyroid tumors are rare endocrine neoplasms affecting 0.1-0.3% of the general population (1); less than 1% of are malignant parathyroid carcinomas (PCs). Age at diagnosis of PCs is commonly in the mid fifth decade of life, usually 10 years earlier than benign parathyroid adenomas (PAs), with no gender prevalence. PC most commonly occurs as a sporadic single-gland disease, without a familial history, with only a minority of cases manifesting as isolated inherited cancer or as part of a complex hereditary syndrome.

The great majority of PCs are functioning tumors over-secreting parathyroid hormone (PTH), responsible for primary hyperparathyroidism (PHPT) and elevated serum levels of calcium; only 2% of PCs are non-functioning variants characterized by normal serum calcium and PTH values, manifesting through compressive signs and symptoms of local invasion (1). Contrary to parathyroid hyperplasia and adenomas, PCs are associated with poor prognosis due to more severely elevated PTH, and higher, commonly unmanageable, hypercalcemia, which accounts for death in a majority of cases, with an overall survival rate of 78-85% and 49-70% at 5 and 10 years after diagnosis, respectively (2). Moreover, 10-30% patients with PC already have metastatic disease at diagnosis, most commonly in the lungs, bone, or liver (3).

The absence of distinctive growth and histological characteristics, as well as of idiosyncratic serum concentration values of calcium ion and intact PTH, distinguishing malignant PCs from PAs, makes a clear pre-operative histopathological or biochemical diagnosis of PCs extremely difficult. A diagnosis of malignancy usually occurs during surgery, through an intraoperative assessment of a marked local invasion of adjacent structures and surrounding lymph nodes, and/or confirmed during a prolonged post-operative follow-up in presence of local relapses or distant metastases. Since PCs require a more radical surgical approach than benign PAs, with *en bloc* gland resection and tumor safety margins, often including the excision of the adjacent ipsilateral thyroid lobe and thymus, a misdiagnosis may lead to inadequate surgical resection and a high recurrence rate and risk of distant metastases. Currently, up to 50% of PC patients manifest metastases following an initial wrong diagnosis of benign disease, with a consequent severe impairment of the prognosis and reduction of overall survival (4).

In this light, the identification of distinctive genetic bases, epigenetic changes, and molecular signatures characterizing parathyroid malignancies is fundamental to identify potential diagnostic biomarkers able to clearly distinguish PCs from benign parathyroid tumors, as well to provide potential therapeutic targets for these malignant neoplasms.

## Genetics of Parathyroid Carcinoma

PCs occur either as sporadic non-hereditary single-gland disease, or as isolated inherited malignancy in a percentage of patients with familial isolated primary hyperparathyroidism (FIPH), or in the context of hyperparathyroidism-jaw tumor (HPT-JT) syndrome, affecting about 15% of HPT-JT patients. Studies on hereditary and sporadic forms of PCs revealed specific germline and somatic genetic alterations underlying PC development (**Table 1**).

**Table 1:** Main genetic determinants in parathyroid carcinomas.

Gene	Gene type in PC carcinogenesis	Chromosomal location	Type of mutations	Encoded protein	Frequency in PCs	Encoded protein function(s)	Molecular and cellular effects of gene mutations
*CDC73* (5, 6)	TSG	1q31.2	Biallelic inactivating mutations/gene loss	Parafibromin	Mutations in 70-100% sporadic PCs (5) 1q31.2 LOH in 50-55% of sporadic PCs (5) Mutations 50-75% of HPT-JT families (5, 6)	Regulation of transcript elongation and stability *via* interaction with the RNA polymerase II. Promotion of histone H3 methylations leading to transcriptionally active chromatin structure. Interaction with the SUV39H1 histone methyltransferase complex and promotion of H3K9 methylation (H3K9me). Interaction with the RNF20/RNF40 ubiquitin-ligase complex and promotion of mono-ubiquitination of the lysine 120 on histone H2B (H2BK120ub1)	Altered expression of genes involved in cell cycle regulation. Transcriptionally inactive conformation of chromatin and inhibition of gene expression. Reduction of H3K9me and subsequent increased transcription of the *CCND1* gene. Altered RNA elongation and gene expression.
*CCND1* (7, 8)	Proto-oncogene	11q13.3	Somatic gene copy amplifications	Cyclin D1	Gene amplification in about 29% of cases (8) Cyclin D1 over-expression in about 90% of cases (7)	Positive regulator of cell cycle progression by promoting the G1 to S phase transition through activation of CDK4 and CDK6.	Over-expression of cyclin D1 protein. Enhanced cell proliferation. Enlarged hyperplastic parathyroid glands. Increased secretion of PTH.
*EZH2* (9)	Proto-oncogene	7q36.1	Somatic gene copy amplifications	EZH2	About 60% of sporadic PCs (9)	EZH2 protein is a histone 3 lysine 27 methyltransferase that promotes the H3K27me3, a histone modification that is commonly associated with transcriptional inhibition.	Increased H3K27me3 across the genome and gene expression repression. Accumulation of the transcriptionally active form of β-catenin, and nuclear transduction of the Wnt signaling.
*PRUNE2* (8, 10)	TSG	9q21.2	Germline and/or somatic biallelic inactivating mutations/gene loss	Prune Homolog 2 with BCH Domain (PRUNE2)	18% of sporadic PCs (10)	Suppression of Ras homolog family member A activity, and subsequent inhibition of oncogenic cell transformation.	Loss of control over cellular transformation.
*AKAP9* (8)	TSG	7q21.2	Somatic biallelic inactivating mutations	A-Kinase Anchoring Protein 9 (AKAP9)	17.6% of sporadic PCs (8)	Member of the A-kinase anchor proteins that regulate cellular localization and functions of the protein kinase A.	The biallelic inactivation suggests the loss of a putative tumor suppressor activity and subsequent loss of the correct cellular localization and function of the protein kinase A.
*ZEB1* (8)	Proto-oncogene	10p11.22	Heterozygote somatic mutations	Zinc Finger E-Box Binding Homeobox 1 (ZEB1)	17.6% of sporadic PCs (8)	A zinc finger transcription factor that acts as a transcriptional repressor, represses E-cadherin promoter, and induces the epithelial-mesenchymal transition (EMT)	Activating mutations are suspected to promote EMT and tumor invasion and metastases.
*ADCK1* (8)	Proto-oncogene?	14q24	A recurrent heterozygote somatic missense mutation	AarF Domain Containing Kinase 1 (ADCK1)	11.8% of sporadic PCs (8)	Still unknown.	Still unknown.
*FAT3* *(* 8)	TSG	11q14.3	Somatic biallelic truncating mutations	FAT Atypical Cadherin 3 (FAT3)	11.8% of sporadic PC (8)	Member of the atypical cadherin family. The exact biological functions have not yet been elucidated.	The biallelic truncating mutations suggest the loss of a putative tumor suppressor activity.
*PIK3CA* (8, 11)	Proto-oncogene	3q26.32	Activating somatic missense mutations	Phosphatidylinositol-4,5-Bisphosphate 3-Kinase Catalytic Subunit Alpha (PIK3CA)	12.5% of sporadic PC (8)	PI3K/AKT/mTOR pathway that regulates cell signaling transduction, cell proliferation, apoptosis, metabolism, and angiogenesis.	Activating mutations of PIK3CA activates the PI3K/AKT/mTOR pathway, resulting in increased cell proliferation.
*MTOR* (8)	Proto-oncogene	1p36.22	Activating somatic missense mutations	Mechanistic Target of Rapamycin Kinase (mTOR)	8.3% of sporadic PC (8)	PI3K/AKT/mTOR pathway that regulates cell signaling transduction, cell proliferation, apoptosis, metabolism, and angiogenesis.	Activating mutations of mTOR activates the PI3K/AKT/mTOR pathway, resulting in increased cell proliferation.

PCs, Parathyroid Carcinomas; TSG, Tumor Suppressor Gene; LOH, Loss of Heterozygosity; CDK4, cyclin-dependent kinases 4; CDK6, cyclin-dependent kinases 6; PTH, Parathyroid Hormone.

Extremely rare cases of malignant PCs have been reported in Multiple Endocrine Neoplasia type 1 (MEN1) syndrome (less than 15 cases) and in Multiple Endocrine Neoplasia type 2A (MEN2A) syndrome (only two cases), as a consequence of germline heterozygote inactivating mutations of the *MEN1* tumor suppressor gene and germline heterozygote activating mutations of the *RET* proto-oncogene, respectively (5). No genotype-phenotype correlation has been found to explain why these few individuals develop PCs instead of PAs, which typically affect patients with MEN1 and MEN2A.

### *CDC73* Gene

Biallelic loss-of-function mutations/inactivation of the Cell Division Cycle 73 (*CDC73*) tumor suppressor gene are the major driver genetic defects in the etiology of PCs and, since they are found in less than 1% of benign PAs (12), they can be considered the main genetic hallmark of parathyroid malignancy risk.

In the hereditary forms (HPT-JT- and FIPH-related cancers), *CDC73* mutations comprise one germline mutation and one somatic inactivation/loss of the gene, the first inherited from the affected parent, and the second occurring in the parathyroid cell, often as chromosomal loss at 1q31.2. Germline inactivating mutations of the *CDC73* gene are detected in 50-75% of HPT-JT families (5, 6), and in about 8% of FIPH pedigrees (13). Over 85% of germline *CDC73* mutations in hereditary PCs are frameshift and nonsense variants that create a premature stop codon, predicting a truncated protein, while the second somatic hit involves loss of heterozygosity (LOH) at the 1q31.2 locus in a great majority of cases.

In sporadic non-inherited PCs, both the *CDC73* loss-of-function mutations occur at somatic level in the parathyroid cell. Somatic *CDC73* mutations have been reported in 40-100% of sporadic PCs (5). Interestingly, germline *CDC73* mutations are identified in 20-40% of patients with apparently sporadic PC (5), suggesting them as probable inherited diseases in those cases where the familial history is unknown, or cases of mosaicism with a germline mutation that originated *de novo* at the embryonic level (14). LOH at the *CDC73* locus is reported in 50-55% of sporadic PCs (5). Hewitt et al. (14) showed hypermethylation of the *CDC73* gene promoter in 18% (2/11) of sporadic PCs and 17% (1/6) of PAs from HPT-JT patients, all without mutation of the *CDC73* gene, but not in 37 sporadic PAs, suggesting this epigenetic gene-silencing tool as an additional mechanism by which loss of the *CDC73* gene expression may give rise to PCs.

**Figure 1:**
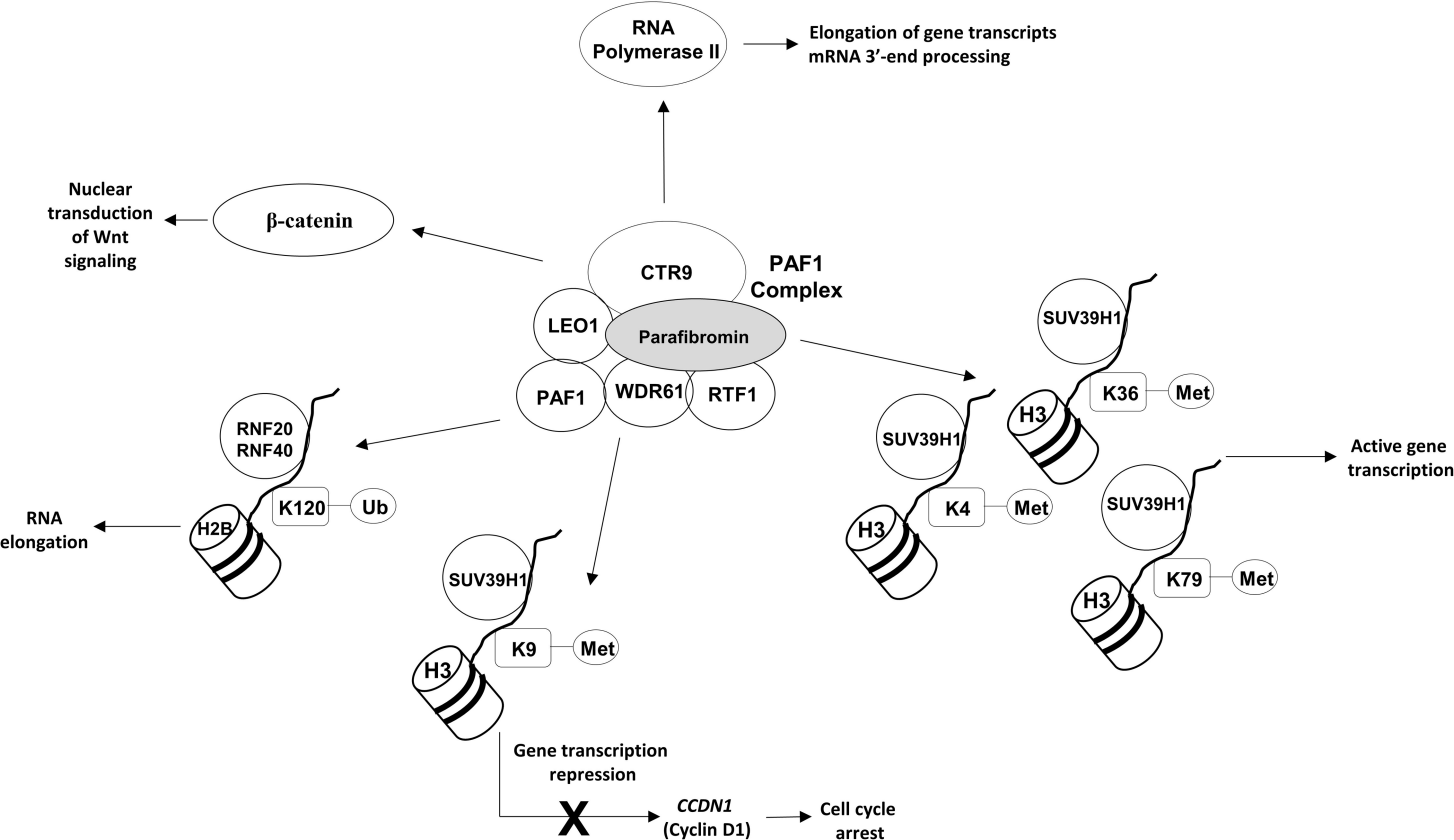
Main roles of parafibromin.

The *CDC73* gene is located on chromosome 1q31.2 and consists of 17 exons encoding parafibromin, a ubiquitously expressed 60 KDa protein that is an essential component of the Polymerase-Associated Factor 1 (PAF1) complex, whose main biological roles are resumed in **Figure 1**.

The PAF1 complex directly interacts with both the non-phosphorylated and the Ser2- and Ser5-phosphorylated forms of the RNA polymerase II large subunit, coordinating the correct recruitment of transcript elongation and RNA processing machineries (15). Moreover, the PAF1 complex is involved in coupling transcriptional and post-transcriptional events, in modulating the transition of gene transcripts from initiation to elongation steps, and in the mRNA 3’-end processing through modulation of the levels of Ser2 phosphorylation in the carboxy-terminal domain (CTD) of the RNA polymerase II (15). The interaction between RNA polymerase II and PAF1 depends on the C-terminal domain of parafibromin, which is deleted in over 80% of clinically relevant mutations of the *CDC73* gene. Loss of parafibromin results in a significant reduction in PAF1 complex binding to RNA polymerase II, in a reduction of Ser2 phosphorylation of the RNA polymerase II CTD, and in shortened poly(A) tails on most cellular transcripts, altering the correct expression of several genes, including genes involved in cell cycle regulation, protein synthesis, and lipid and nucleic acid metabolism (16).

In the absence of parafibromin, replication-dependent histone mRNAs are not cleaved and contain poly(A) tails, being more stable. In sporadic PCs, loss of parafibromin has been associated with over-expression of some members of the replication-dependent histones family, H1.2, H2A and H2B, whose genes belong to a histone micro-cluster on chromosome 6p22.2-p21.3 (17). H1.2 inhibits transcription of growth suppressive genes *via* modulation of chromatin structure (18), thus, the H1.2 increased expression may result in a promotion of cell proliferation.

The PAF1 complex is also involved in multiple aspects of histone modifications (**Figure 1**). It promotes histone 3 methylations on lysine residues 4, 36 and 79 (H3K4me, H3K36me and H3K79me), which are generally associated with transcriptionally active chromatin (16). PAF1 has also been demonstrated to promote histone 3 methylation on lysine 9 (H3K9me) (19) through a direct interaction of the central region of parafibromin (amino acids 128–250) with SUV39H1 histone methyltransferase complex. Amino acids 128-227 have been demonstrated to be the minimal necessary for functional interaction with SUV39H1 (19). PAF1-induced H3K9me results in a transcriptional repression of the *CCDN1* gene, encoding cyclin D1, and in a subsequent cell cycle arrest at the G1 to S phase transition. Moreover, PAF1 promotes the histone H2B mono-ubiquitination on the lysine residue 120 (H2BK120ub1), a histone modification that is positively involved in RNA elongation through a direct interaction with the RNF20/RNF40 ubiquitin-ligase complex (20).

The PAF1 complex has been demonstrated to be an essential regulator of the nuclear transduction of the Wnt signal *via* a direct protein-protein interaction between parafibromin, LEO1, and β-catenin. Parafibromin has been shown to directly bind to the C-terminal region of the β-catenin through the evolutionarily conserved central region of the parafibromin protein (amino acids 218-263), which itself has been found to be sufficient for potent β-catenin binding (21). The exact role of parafibromin in Wnt signaling has not been clearly established yet, and it might vary by cell type.

Parafibromin is located prevalently in the nucleus, thanks to a nuclear localization signal (amino acids 125-136) and a nucleolar localization signal (amino acids 76-92); loss of one or both of these domains, following *CDC73* truncating mutations, prevents the nuclear localization of parafibromin and its main functions, and it has been associated with HPT-JT syndrome (22, 23). Loss of parafibromin nuclear immunostaining has been found in PCs and appears to be a distinctive histological feature characterizing malignant tumors from benign parathyroid tumors (24). Both sporadic and HPT-JT-related PCs with biallelic mutation/loss of the *CDC73* gene have shown loss of nuclear localization and activity of parafibromin that were strongly associated with tumor malignant behavior, with a younger age of patients and larger tumor size with respect to other *CDC73*-positive PCs (12). Moreover, PCs negative for parafibromin nuclear immunostaining have shown distinctive morphological (i.e., eosinophilic cytoplasm, very frequent presence of perinuclear cytoplasmic clearing, nuclear atypia, and nuclear enlargement with distinctive coarse chromatin) and proliferative (extensive sheet-like growth rather than acinar architecture) features, which suggest parafibromin-deficient parathyroid malignancies to be a distinct histological and clinical subtype of PCs (12).

### *CCND1* Gene

Cyclin D1 is a positive regulator of cell cycle progression that specifically promotes the G1 to S phase transition of the cell cycle through the activation of the cyclin-dependent kinases 4 and 6 (CDK4 and CDK6). Cyclin D1 is encoded by the *CCND1* proto-oncogene, located on chromosome 11q13.3. The over-expression of cyclin D1 protein is an extremely common event in parathyroid carcinogenesis, characterizing about 90% of PCs (7).

A direct pathogenic role of cyclin D1 in parathyroid tumorigenesis has been demonstrated in transgenic mouse models containing a chromosomal rearrangement of the human *CCND1* locus, mimicking the one found in two human parathyroid tumors, and over-expressing cyclin D1. As a consequence of cyclin D1 over-expression, mutated mice developed enlarged hyperplastic parathyroid glands, characterized by increased cell proliferation and enhanced PTH secretion, and manifested a chronic PHPT with hypercalcemia and bone abnormalities (25). In these mice, the cyclin D1-induced abnormal parathyroid proliferation temporarily preceded the dysregulation of the calcium-PTH axis, confirming the direct action of up-regulated cyclin D1 on enhancement of parathyroid cell growth, and indicating that the disturbed parathyroid proliferation is the crucial primary initiator leading to the PHPT phenotype (26).

The exact mechanisms responsible for cyclin D1 over-expression in parathyroid carcinogenesis are still largely unclear. As for other types of human cancers, over-expression of cyclin D1 in parathyroid tumor cells presumably derives from gene copy number alterations or chromosomal rearrangements and trans-acting altered regulation of gene expression, rather than activating mutations of the *CCDN1* gene. A heterozygote p15-q13 pericentromeric inversion in chromosome 11 was reported in two unrelated cases of benign PAs, positioning the 5’-regulatory element of the PTH gene from the 11p15 locus directly upstream of the *CCDN1* gene on the 11q13 locus, inducing over-expression of cyclin D1 protein, and, at the same time, maintaining one intact copy of the *PTH* gene and, thus, the capability to express PTH (27). However, this chromosomal translocation has not been described in PCs. A whole-genome study on sporadic PCs showed somatic amplification of the genomic region containing the *CCDN1* gene in 29% of analyzed cases, 80% of them being mutually exclusive with cases harboring *CDC73* somatic mutations (8), suggesting that *CCDN1* amplification is an alternative genetic mechanism to *CDC73* inactivation to up-regulate cyclin D1 expression. Since cyclin D1 up-regulation is a frequent event also in benign sporadic PAs (20-40% of cases), the over-expression of this protein cannot be used as a hallmark to distinguish benign from malignant tumors.

Over-expression of cyclin D1 in mouse fibroblasts has been associated with an enhanced and more rapid cell cycle progression, due to a shortened duration of the G1 phase, and with the induction of anchorage-independent growth and enhanced invasion ability (28). At the molecular level, up-regulated cyclin D1 led to the activation of the pRB/E2F pathway, a pathway that has been shown to be deregulated in a large majority of human tumors, and the consequent increased expression of Fibroblast Growth Factor Receptor 1 (FGFR1), both at the mRNA and protein levels, which presumably acts as a direct mediator of the increased cell invasion of the matrigel in response to treatment with basic Fibroblast Growth Factor (bFGF). These data suggest that the pro-oncogenic action of up-regulated cyclin D1 can be mediated, at least in part, by potentiating the stimulatory effects of bFGF, a pro-proliferation factor that is often produced by stromal cells, on the growth and invasiveness of adjacent tumor cells (28), a mechanism that can also be suspected in parathyroid cancer.

### *PRUNE* Gene

The Prune Homolog 2 with BCH Domain (*PRUNE2*) gene was recently identified as a novel PC-associated gene (10). The protein encoded by *PRUNE2* gene belongs to the B-cell CLL/lymphoma 2 and adenovirus E1B 19 kDa interacting family, whose members play roles in many cellular processes, including apoptosis and cell transformation. PRUNE2 protein is a tumor suppressor exerting various biological functions, including the suppression of Ras homolog family member A activity, which results in inhibition of oncogenic cellular transformation.

A whole-exome sequencing analysis on PCs showed recurrent mutations of the *PRUNE2* gene in 18% of cases (10). No mutation was detected in the 40 PA validation set, suggesting *PRUNE2* gene as a novel candidate gene in malignant carcinogenesis and a possible genetic determinant to diagnose PCs. A germline missense mutation (p.Val452Met) has been identified in a PC sample in absence of *CDC73* mutation and with a somatic LOH at the chromosome 9, containing the *PRUNE2* gene. Two somatic nonsense mutations (c.Glu474X and p.Glu537X), presumably inactivating both the two gene alleles, were found in a PC sample bearing a concomitant *CDC73* mutation, suggesting a possible synergic effect of these two mutated tumor suppressor genes in determining malignant cancer development. No data were available on the clinical characteristic of that PC, indicating a more severe cancer phenotype as a consequence of the presence of double-gene mutations. These two stop-gain mutations were confirmed as possible causes of PC development by another genome-sequencing study, and both were found at somatic levels in one single PC sample, but not in the corresponding normal parathyroid tissue (8). Two somatic missense mutations (p.Gly455Asp and p.Ser450Asn) were identified in two PC cases, both without *CDC73* and *MEN1* mutations.

All three identified missense mutations were predicted to be probably damaging and likely pathogenic by disrupting the function of the PRUNE2 protein. The two nonsense mutations were predicted to produce a truncated PRUNE2 protein lacking the BCH domain and, thus, presumably losing the overall tumor suppressor functions, leading to loss of control over cellular transformation. A study by Yu et al. (10) performed a whole-gene sequencing on 8 PCs included in the discovery cohort, localizing all the three identified mutations of the *PRUNE2* gene in the exon 8, which appeared to be a possible mutational hot spot. As a consequence, the additional 13 PC samples of the validation cohort were sequenced only for exon 8, presumably leading to an underestimation of the real *PRUNE2* mutation rate in PCs. Whole *PRUNE2* gene sequencing on larger and different series of PC patients is needed to confirm *PRUNE2* mutations as a genetic cause of malignancy of parathyroid glands and to identify a possible occurrence of mutations in other regions of the gene.

### *EZH2* Gene

The Enhancer of Zeste Homolog 2 (*EZH2*) gene encodes a histone 3 lysine 27 methyltransferase that is a member of the Polycomb Repressive 2 (PCR2) complex. *EZH2* gene amplification at 7q36.1 locus is common in PCs (about 60% of cases), acting as an oncogene in parathyroid carcinogenesis and showing a significant over-expression of *EZH2* mRNA and protein in malignant carcinomas compared with adenomas and hyperplastic parathyroid glands (9). Conversely, no *EZH2* gene mutations were found in sporadic PCs (29), while a recurrent p.Tyr641Asp missense mutation was identified in unrelated PAs (30, 31). Therefore, different types of genetic aberrations of the *EZH2* gene appear to differentially characterize PCs and PAs.

**Figure 2:**
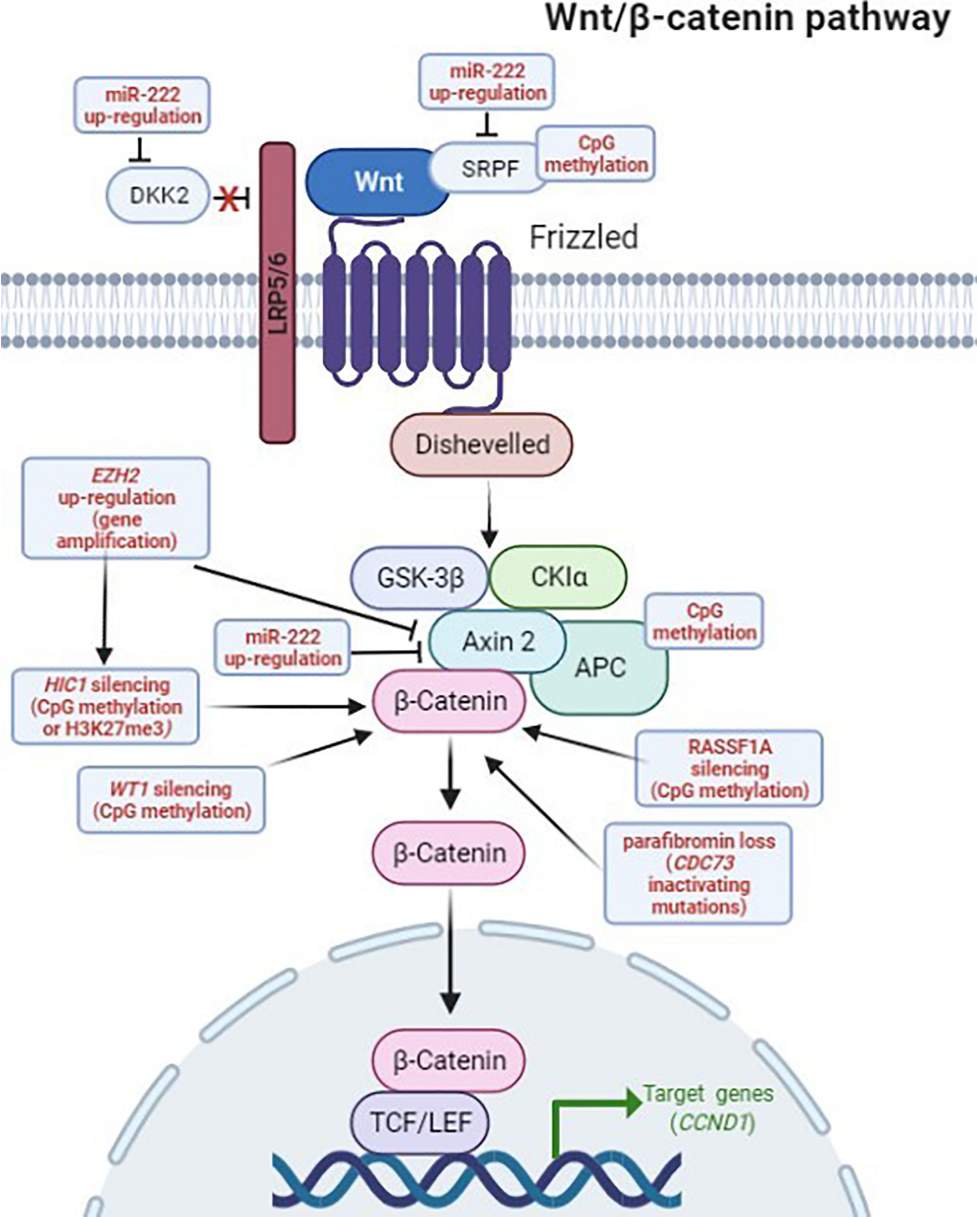
Deregulation of the Wnt/β-catenin signaling in PCs. Genetic and epigenetic aberrations detected in PC, altering the Wnt/β-catenin pathway, are highlighted in red. In the canonical Wnt/β-catenin pathway, the Wnt ligands initiate signaling by interacting, on the cell surface, with the Frizzled (FZD) receptor and the low density lipoprotein receptor-related protein 5/6 (LRP5/6) coreceptor. The ligand-activated FZD-LRP5/6 complex recruits, respectively, Dishevelled and AXIN2 intracellular proteins, preventing the constitutive destruction of cytosolic β-catenin by disassembling the β-catenin destruction complex, consisting of AXIN2, adenomatous polyposis coli (APC), glycogen synthase kinase3β (GSK3β) and casein kinase 1 (CK1), and ultimately leading to active β-catenin accumulation and nuclear translocation. In the nucleus, β-catenin binds and activates transcription factors of the lymphoid enhancer-binding factor (LEF)/T-cell factor (TCF) family, leading to the transcription of Wnt target genes (i.e. *CCND1*). DKK2 and SFRP proteins are natural extracellular inhibitors of the Wnt signaling, by respectively binding, and inactivating, the LRP5/6 coreceptor and the Wnt ligands. Figure created in BioRender.com, accessed on 10 January 2022.

Increased expression and enzymatic activity of EZH2 protein result in an elevated histone trimethylation on lysine 27 (H3K27me3) across the genome, a histone modification that is commonly associated with transcriptional inhibition. A suspected target gene, repressed by EZH2-mediated H3K27me3, is the *HIC1* tumor suppressor gene, which is normally involved in control of parathyroid growth; an aberrant down-regulation of *HIC1* has been observed in both benign and malignant parathyroid, presumably due to the repressive action of H3K27me3 on gene expression (32) (**Figure 2**).

Normally, EZH2 mediates the epigenetic gene transcription repression of several Wnt antagonists, including the growth-suppressive AXIN2 protein, a negative regulator of cytoplasmatic levels and stability of β-catenin in the absence of Wnt ligand. As a consequence, an excessive expression of EZH2 represses AXIN2, and it has been demonstrated to significantly contribute to an aberrant accumulation of the transcriptionally active (non-phosphorylated) form of β-catenin in the cytoplasm and the consequent nuclear translocation, resulting in the nuclear transduction of Wnt signaling that leads to increased cyclin D1 expression and enhanced cell proliferation (9) (**Figure 2**).

### *ADCK1* Gene

In a whole-genome sequencing analysis, a recurrent missense mutation p.Ile482Met in the *ADCK1* gene, encoding the AarF Domain-Containing Kinase 1 (ADCK1), was found in 2 of the 17 analyzed PC cases (11.8%) (8). The role of the ADCK1 protein is not yet clear; it is not known if it has a protein kinase activity or which amino acid residue it would phosphorylate (Ser, Thr or Tyr). The monoallelic somatic *ADCK1* mutation suggests a dominant-negative effect of the mutated protein and the *ADCK1* gene as a candidate oncogene in PC development.

### *AKAP9* Gene

A whole-genome sequencing analysis on 17 sporadic PC samples has identified the *AKAP9* gene as recurrently mutated (3/17; 17.6% of analyzed cases) (8). The *AKAP9* gene encodes the A-Kinase Anchoring Protein 9 (AKAP9), a member of the A-kinase anchor proteins, that regulates cellular localization and function of the protein kinase A. Two missense and one nonsense somatic mutations were identified in 3 cases, two of them showing the biallelic inactivation of the gene, thus suggesting a tumor suppressor activity of *AKAP9* in parathyroids. Further functional investigations are needed to assess the potential role of *AKAP9* loss/inactivation as oncogenic driver in PC development.

### *ZEB1* Gene

The *ZEB1* gene encodes the Zinc Finger E-Box Binding Homeobox 1 (ZEB1) protein, a zinc finger transcription factor that acts as a transcriptional repressor, represses E-cadherin promoter and induces the epithelial-to-mesenchymal transition (EMT), promoting tumor invasion and metastases.

Somatic heterozygote mutations of the *ZEB1* gene have been recurrently identified in PC samples (17.6% of cases analyzed by a whole-genome sequencing) (8). The molecular and cellular effects of the identified *ZEB1*mutations in PCs have not yet been evaluated, but activating mutations are suspected to promote EMT and consequent tumor invasivity and metastatic capability.

### *FAT3* Gene

The *FAT3* gene has recently been recognized as a possible candidate gene in sporadic PC development by whole-genome profiling (8). This gene encodes the FAT Atypical Cadherin 3 (FAT3) protein, whose biological role has not yet been clearly defined. FAT3 belongs to a family of proteins that includes two known tumor suppressors, FAT1 and FAT4.

Two different somatic nonsense mutations were identified in 2/17 (11.8%) analyzed cases, both with the biallelic inactivation of the gene and creating a premature stop codon near to the N-terminal region of the FAT3 protein, suggesting the *FAT3* gene as a putative tumor suppressor gene in parathyroid carcinogenesis, possibly regulating the Wnt signaling.

### PI3K/AKT/mTOR Signaling Pathway

The PI3K/AKT/mTOR pathway is an intracellular signaling pathway important for regulating cell signal transduction and biological processes, such as cell proliferation, apoptosis, metabolism, and angiogenesis. Several studies have shown that the deregulation of this signaling pathway is involved in the occurrence and development of different types of human malignancies (33). A genomic profiling of 24 PCs revealed the presence of somatic activating missense variants in genes encoding components of this signaling pathway in 21% (5/24) of cases: p.Lys111Glu, p.Glu545Ala, and p.His1047Arg in the *PIK3CA* gene, and p.Leu1460Pro and p.Gln2524Leu in the *MTOR* gene (8). Results from this study suggest the altered PI3K/AKT/mTOR pathway as a major oncogenic factor in parathyroid carcinogenesis.

The somatic activating missense p.Glu545Lys variant of the *PIK3CA* gene was identified, through a complete genome landscape mutational analysis of tumor specimens, in a primary sporadic PC, but not in the 7 PC recurrences, suggesting a role for mutated PIK3CA protein in tumor initiation, but not in tumor progression and tissue invasion (11).

### *YAP1* Gene

The Yes Associated Protein 1 (*YAP1*) gene encodes a downstream nuclear effector of the Hippo signaling pathway, which is known to play a role in the development and progression of multiple cancers, by acting as a transcriptional regulator of this signaling pathway. Recently Tavanti et al. (34) found a remarkable loss of YAP1 protein nuclear staining in formalin-fixed paraffin-embedded PC specimens, compared with normal parathyroid and PA samples that were both characterized by a positive nuclear expression of YAP1. Loss of YAP1 nuclear localization was independent of *CDC73* and *MEN1* gene status. No data were reported about *YAP1* gene status at both germline and somatic levels in PCs, and the molecular mechanism responsible for loss of YAP1 nuclear localization in PC remains to be fully elucidated.

### *FLNA* Gene

The filamin A (*FLNA*) gene encodes the homonym filamin A protein, a non-muscle actin filament cross-linking scaffold protein that influences the intracellular localization of numerous proteins. A very recent study by Storvall et al. (35) found that PCs were characterized by significantly increased cytoplasmic FLNA expression, compared to both PAs and atypical parathyroid adenomas (aPAs), suggesting that the increased cytoplasmic, and membranous, FLNA expression could be a marker of malignancy, and a potential prognostic predictor of aggressive behavior in parathyroid neoplasms, mostly in combination with parafibromin expression. However, a previous study (36) showed opposite results, reporting a decreased expression of full-length FLNA in the cytoplasm of tumoral parathyroid epithelial cells, both for PAs (50% to 10% of FLNA-positive cells) and PCs (less than 10% or totally absent FLNA-positive cells), with respect to the normal parathyroid counterpart.

Therefore, the role of FLNA expression levels in parathyroid carcinogenesis needs to be clearly elucidated by further studies.

### *TBX1* Gene

A greater than two-fold reduced expression of the T-Box Transcription Factor 1 (*TBX1*) gene, with respect to normal parathyroids, was found in 71% (10/14) of human PCs at mRNA level, and confirmed, at the protein level, by in a significant decrease of TBX1-positive immunostained cells (37). Interestingly, in all the 10 PC samples characterized by a reduced number of TBX1-expressing cells, this molecular signature was associated with both the downregulation of *TBX1* transcript and the loss of parafibromin immunostaining, while the 4 PC samples conserving parafibromin expression had *TBX1* mRNA expression levels similar to those of normal parathyroid, suggesting a regulatory relationship between TBX1 and parafibromin, involved in parathyroid carcinogenesis.

## Epigenetics of Parathyroid Carcinoma

Epigenetic processes are one of the most important regulators of gene expression and one of the most often deregulated mechanisms in human tumors. Epigenetic mechanisms are heritable but reversible, and they include DNA methylation, histone modifications, and small and long non-coding RNAs.

### DNA Methylation in Parathyroid Carcinoma

DNA methylation consists of the covalent addition of a methyl group at the 5’-position of a cytosine, usually followed by a guanine (CpG dinucleotide). DNA methylation is catalyzed by DNA methyltransferases (DNMTs), while DNA demethylation is promoted by ten-eleven translocation (TET) methylcytosine dioxygenases. CpG dinucleotides are spread out across the genome, being commonly heavily methylated, with the exception of CpG islands, which are prevalently located in the promoter regions of genes. Global CpG methylation in intergenic regions is a fundamental mechanism to repress the expression of potentially harmful genetic sequences, such as transportable and retroviral elements. The majority of human gene promoters (roughly 70%) reside within CpG islands; the hypermethylation of these CpG islands is a reversible mechanism of gene silencing, responsible for regulating tissue-specific gene expression.

Human cancers are generally characterized by a global DNA hypomethylation, with respect to their normal counterparts. In contrast, both benign and malignant parathyroid tumors show a global methylation pattern similar to normal parathyroid glands (38, 39).

A genome-wide analysis of CpG island methylation identified 367 gene promoters significantly differentially methylated between PAs and normal parathyroids, 175 between PCs and normal parathyroids and 263 between PAs and PCs (40). In-depth analysis of the top 100 differentially methylated CpG islands showed, in PC samples, the presence of hypermethylation of all the examined sites (40).

Promoter hypermethylations of Adenomatous Polyposis Coli (*APC*) and Ras-association Domain Family Member 1A (*RASSF1A*) tumor suppressor genes are two common epigenetic changes in parathyroid tumors (38–41), which have been interestingly found in the 100% of analyzed PC samples (39, 41) (**Table 2**). Both silencing of *APC* and *RASSF1A* genes lead to the accumulation of the active form of β-catenin and to the subsequent activation of the canonical Wnt signaling, resulting in transcription of TCF/LEF-responsive target genes, including *CCND1.*

Table 2Hypermethylated gene promoters in parathyroid tumors compared to normal parathyroid tissue.

**Table:** 

Hypermethylated gene promoters in parathyroid carcinomas					
Gene	Encoded protein	Encoded protein function(s)	Frequency in PCs		Molecular and cellular effects of promoter hypermethylation-driven gene silencing
*HIC1* (32)	HIC ZBTB Transcriptional Repressor 1 (HIC1)	Transcription repressor that inhibits expression of E2 transcription factor 1 (E2F1) by directly binding its promoter, and positively modulates p53 function by repressing transcription of the deacetylase SIRT1 that can inactivate p53 by deacetylation. HIC1 negatively regulates the Wnt pathway and the TCF-mediated gene transcription by directly binding TCF4 and β-catenin and preventing them from binding to the promoters of the TCF-responsive genes.	100% (5/5) of sporadic PCs (32)		Silencing of the *HIC* gene may result in: - reduction/loss of p53 tumor suppressor activity; - activation of the canonical Wnt/β-catenin pathway with transcription of TCF-responsive genes (**Figure 2**).
*PYCARD* (40)	PYD and CARD Domain Containing (PYCARD)	PYCARD is an adaptor protein, composed of two protein-protein interaction domains, that mediates assembly of large signaling complexes in apoptotic signaling pathways *via* the activation of caspase.	Not reported		Silencing of the *PYCARD* gene may play an important role in escaping from apoptosis in parathyroid carcinogenesis.
*GATA4* (40)	GATA Binding Protein 4 (GATA4)	GATA4 is a zinc-finger transcription factor that recognizes and binds the GATA motif present in the promoters of many genes, regulating gene expression.	Not reported		Not defined in parathyroid tumorigenesis.

**Table:** 

Hypermethylated gene promoters both in parathyroid carcinomas and parathyroid adenomas.					
Gene	Encoded protein	Encoded protein function(s)	Frequency in PCs	Frequency in PAs	Molecular and cellular effects of promoter hypermethylation-driven gene silencing
*RASSF1A* (38, 39)	Ras Association Domain Family Member 1 (RASSF1A)	A tumor suppressor gene suspected to regulate cell proliferation and apoptosis.	100% (3/3) of sporadic PCs (39)	98% (54/55) of sporadic PAs (38) 52% (34/66) of sporadic Pas (38)	Silencing of the *RASSF1* gene leads to β-catenin accumulation, activation of Wnt-regulated gene transcription and enhanced cell growth (**Figure 2**).
*APC* (39)	Adenomatous Polyposis Coli Protein (APC)	APC is a tumor suppressor acting as a negative regulator of the canonical Wnt signaling pathway by promoting degradation of β-catenin.	100 (5/5) of sporadic PCs (41)	56% (37/66) of sporadic Pas (39)	Silencing of the *APC* gene leads to deregulated activation of the canonical Wnt/β-catenin pathway and enhanced cell growth (**Figure 2**). Treatment of a primary PC cell culture with a DNA hypomethylating agent (decitabine) restored APC expression, reduced active non-phosphorylated β-catenin, inhibited cell growth, and induced apoptosis, confirming the role of *APC* promoter hypermethylation in promoting an aberrant cell growth in the parathyroids (41).
*SFRP1* (40)	Secreted Frizzled Related Protein 1 (SFRP1)	Inhibitor of the Wnt/β-catenin pathway.	Not reported	Not reported	Epigenetic silencing of SFRP genes leads to deregulated activation of the canonical Wnt/β-catenin pathway, which is associated with cancer (**Figure 2**).
*SFRP2* (40)	Secreted Frizzled Related Protein 2 (SFRP2)	Inhibitor of the Wnt/β-catenin pathway.	Not reported	Not reported	Epigenetic silencing of SFRP genes leads to deregulated activation of the canonical Wnt/β-catenin pathway, which is associated with cancer (**Figure 2**).
*SFRP4* (40)	Secreted Frizzled Related Protein 4 (SFRP4)	Inhibitor of the Wnt/β-catenin pathway.	Not reported	Not reported	Epigenetic silencing of SFRP genes leads to deregulated activation of the canonical Wnt/β-catenin pathway, which is associated with cancer (**Figure 2**).
*CDKN2B* (40)	Cyclin Dependent Kinase Inhibitor 2B (p15^INK4b^)	p15^INK4b^ is a cyclin-dependent kinase inhibitor, which forms a complex with CDK4 or CDK6 and prevents the activation of the CDK kinases, acting as a negative regulator of cell growth by inhibiting cell cycle G1 progression.	Not reported	Not reported	Silencing of the *CDKN2B* gene increases cell proliferation.
*CDKN2A* (40)	Cyclin Dependent Kinase Inhibitor 2A (p16^INK4a^)	p15^INK4b^ is a cyclin-dependent kinase inhibitor, which forms a complex with CDK4 and prevents the activation of the CDK kinases, acting as a negative regulator of cell growth by inhibiting the cell cycle G1 progression.	Not reported	Not reported	Silencing of the *CDKN2A* gene increases cell proliferation
*WT1* (40)	Wilms Tumor Protein (WT1)	WT1 is a transcription factor that plays an important role in cell development and cell survival.	Not reported	Not reported	Silencing of the *WT1* gene eliminates its tumor suppressor activity.
*PRDM2/RIZ1* (42)	PR/SET Domain 2 (PRDM2)	PRDM2 is a zinc-finger protein, member of a nuclear histone/protein methyltransferase superfamily, which plays a role in transcriptional regulation.	100% (1/1) (42)	40% (15/38) (42)	Silencing of the *PRDM2/RIZ1* gene is suspected to eliminate its tumor suppressor activity.

**Table:** 

Hypermethylated gene promoters in parathyroid adenomas.					
Gene	Encoded protein	Encoded protein function(s)	Frequency in PAs		Molecular and cellular effects of promoter hypermethylation-driven gene silencing
*CTNNB1* (39)	β-catenin	β-catenin is a key downstream component of the canonical Wnt signaling pathway.	29% (19/66) of sporadic Pas (39)		Not defined in parathyroid tumorigenesis.
*PAX1* (43)	Paired Box 1 (PAX1)	PAX1 is a transcription factor with a suspected tumor suppressor activity.	35% (14/40) of sporadic Pas (43)		Silencing of the *PAX1* gene is suspected to eliminate its tumor suppressor activity.

PCs, Parathyroid Carcinomas; PAs, Parathyroid Adenomas.

Hypermethylation of promoters of *HIC1*, *PYCARD*, and *GATA4* gene has specifically found to characterize PCs, with respect to both normal parathyroid glands and PAs (32, 40).

Main hypermethylated gene promoters identified in parathyroid tumors are reported in **Table 2**.

### Histone Modifications in Parathyroid Carcinoma

Histones are the protein component of chromosomes, exerting a key role in the packing of chromatin and the regulation of gene expression. These proteins undergo post translational modifications, regulated by specific histone-modifying enzymes, consisting in the addition or removal of one or more methyl, acetyl, phosphate, or ubiquitin groups to specific amino acid residues. Histone modifications are reversible epigenetic mechanisms that alter chromatin state, modifying the accessibility of transcription factors to the promoters of target genes, thus positively or negatively regulating transcriptional activity.

As described above, one of the most commonly lost tumor suppressors in sporadic and syndromic PCs, the parafibromin, is a key regulator of various histone modifications (**Figure 1**). Normally, this nuclear tumor repressor interacts with the SUV39H1 histone methyltransferase complex, promoting H3K4me and H3K79me, and with the RNF20/RNF40 ubiquitine ligase complex, promoting H2BK120ub1.

Two other genes, *EZH2* (9) and *PRDM2/RIZ1* (42), whose expression has been reported to be deregulated in human PCs, respectively by gene amplification and promoter hypermethylation, encode methyl transferases, indicating a role of deregulated histone methylation in parathyroid carcinogenesis.

### Deregulated MicroRNAs in Parathyroid Carcinoma

MicroRNAs (miRNAs) are single-stranded non-coding small RNAs (19-25 nucleotides in length), that negatively regulate the expression of target mRNAs, through a base-pairing mechanism, which leads to transcript endonucleolytic cleavage and degradation, or to transcriptional repression. Over 2,500 different mature human miRNAs have been identified thus far (www.mirbase.org), encoded by about 1,800 genes, which in more than 50% of cases are assembled in genomic clusters, rather than being casually distributed along the genome.

Deregulation of miRNA expression/activity has been found in many types of human cancers, acting as oncogene (oncomiR) or tumor suppressors; tumor cells show a larger alteration of miRNA expression compared to their normal counterparts.

Interestingly, deregulated signatures in miRNA expression appear to specifically characterize PCs, compared to both PAs and normal parathyroid glands, suggesting the possibility of using these epigenetic changes as markers to distinguish different parathyroid tumor types. PCs show a miRNA global down-regulation (about 80%) with respect to normal parathyroids. The most significantly down-regulated miRNAs in PCs were miR-26b (44), miR-30b (44), miR-126-5p (44), and miR-296-5p (45), while significantly up-regulated miRNAs were miR-222 (45), miR-517c (46), and miR-503 (45). Conversely, miR-139-3p resulted similarly expressed in PCs and PAs, and significantly down-regulated in both parathyroid tumor types compared to healthy glands (45).

Deregulated miRNAs specifically characterizing PC, and their possible role in carcinogenesis, are reported in **Table 3**.

**Table 3:** Deregulated miRNAs and lncRNAs in parathyroid carcinomas.

Deregulated miRNAs in parathyroid carcinomas				
miRNA[reference]	Variation in PCs	Target mRNA(s)	Biological function of targeted mRNA(s)	Effect of miRNA deregulation in PCs
miR-26b (44)	Down-regulated	PTEN	The *PTEN* gene is a tumor suppressor encoding the phosphatidylinositol-3,4,5-trisphosphate 3-phosphatase protein (PTEN), which negatively regulates the intracellular levels of phosphatidylinositol-3,4,5-trisphosphate and the AKT/PKB pathway, involved in promoting cell survival and growth in response to extracellular signals.	No studies available.
miR-30b (44)	Down-regulated	TRIM27	Tripartite motif−containing 27 (TRIM27) is a component of the TRIM27-PI3K/Akt axis, involved in various malignant tumor processes, such as promotion of cell proliferation, inhibition of apoptosis, and facilitation of cell invasion and metastases. TRIM27 has been shown to function as an oncogene by activating epithelial-mesenchymal cell transition and its up-regulation has been associated with tumor invasion, metastasis and prognosis.	No studies available
miR-126-5p (44)	Down-regulated	EGFL7	The *EGFL7* gene encodes the epidermal growth factor-like domain 7 (EGFL7) protein that is involved in cellular migration and angiogenesis.	No studies available.
miR-296-5p (45)	Down-regulated	HGS	The encoded HSG protein plays a critical role in degradation and recycling of membrane receptors by sorting monoubiquitinated membrane proteins into exosomes and targeting them for lysosome-dependent degradation.	In human PC samples, the down-regulation of miR-296-5p resulted in increased HGS mRNA levels. A dramatic immunostaining-detected over-expression of HGS protein was observed in PCs, compared with both PAs and normal glands (45).
miR-222 (45)	Up-regulated	CDKN1B	The *CDKN1B* gene encodes the p27^/kip1^ protein, a cyclin-dependent kinase inhibitor, which negatively regulates cell cycle progression and cell growth.	In human PC samples, the over-expression of miR-222 resulted in almost complete loss of expression and nuclear localization of the p27^/kip1^ protein (45).
miR-517c (46)	Up-regulated	Still unknown	Non applicable.	No studies available.
miR-503 (45)	Up-regulated	CCDN1	The *CCDN1* gene encodes cyclin D1, a positive regulator of cell cycle progression that promotes the G1 to S phase transition through activation of CDK4 and CDK6.	In human PC samples with up-regulation of miR-503, cyclin D1 displayed a heterogeneous immunoreactivity (42). High levels of cyclin D1 were also found in a subset of PAs, seeming to exclude miR-503 as a main modulator of cyclin D1 expression in parathyroid cells.
**Deregulated lncRNAs in parathyroid carcinomas**				
**LncRNA**	**Variation in PC**	**Biological function of lncRNA**		**Effects of lncRNA deregulation in PC**
lncRNA GLIS2-AS1 (47)	Down-regulated	Still unknown		No studies available.
lncRNA PVT1 (47)	Up-regulated	The *PVT1* gene has been identified as a candidate oncogene. Increased copy number and over-expression of this gene have been associated with many types of human cancers.		No studies available.
lncRNA BC200 (48)	Up-regulated	lncRNA BC200 is a protein-interacting non-coding RNA, presumably involved in the regulation of translation repression.		No studies available.

PC, Parathyroid Carcinoma; PA, Parathyroid Adenoma.

Among deregulated miRNAs, miR-517c, located within the C19MC miRNA cluster at 19q13.42, showed the most significant expression difference between PC and PA, and its expression in PA was similar to that observed in normal parathyroids, strongly suggesting this miRNA as a potential diagnostic biomarker of PC, and potentially involved in determining the malignant phenotype. In PC samples, the up-regulation of miR-517c significantly correlated with over-expression of miR-371 and miR-372, two miRNAs of the miR-371-373 cluster, closely distal to C19MC. Deregulated expression profiles of miR-517c, miR-371, and miR-372, observed in primary PC, were conserved in their matched metastases (46). Considering clinical features of PC patients, expression levels of miR-517c in PC samples positively correlated with serum values of calcium and PTH, and with tumor weight (46). MiR-517c showed a tumor suppressor activity in human glioblastoma (49) and hepatocellular carcinoma cells (50), respectively through the inhibition of the epithelial-to-mesenchymal-like transition phenotype by targeting KPNA2 mRNA and, thus, disrupting the TP53 nuclear translocation, and through the inhibition of cell proliferation by targeting PTK2B/Pyk2 mRNA. Conversely, in PC, miR-517c appears to act as a proto-oncogene, although the pro-oncogenic effect of miR-517c up-regulation and the target mRNA(s) in parathyroid glands are not yet known.

Down-regulation of miR-296-5p showed a valuable predictive value in distinguishing PCs from healthy parathyroids (45); this miRNA was also down-regulated in a lung metastasis from a primary PC (46). MiR-296-5p is located at the imprinted complex locus GNAS (encoding the alpha subunit S of the guanine nucleotide-binding protein) on chromosome 20q13 and is expressed from the paternally-inherited allele (GNAS antisense RNA1; *GNAS-AS1*), but not from the maternally-derived allele (51). Interestingly, down-regulation of miR-296-5p seems to be restricted to PC, and it has not yet been reported in other human malignancies, indicating this miRNA as a possible parathyroid tissue-specific tumor suppressor. The putative mRNA target of miR-296-5p, the Hepatocyte Growth Factor-Regulated Tyrosine Kinase Substrate (HGS), which has been found to be over-expressed in PC samples with down-regulated miR-296-5p, is involved in the Wnt pathway, which is often altered in parathyroid tumors, suggesting a pro-oncogenic role of HGS up-regulation in parathyroid glands *via* Wnt signaling.

Interestingly, miR-222 was shown to suppress the expression of multiple negative regulators of the Wnt/β-catenin pathway, including DKK2, WIF2, SFRP2 and AXIN2 (**Figure 2**) (52). This miRNA is over-expressed in the majority of epithelial tumors, where it decreases the expression of epithelial-specific genes and increases the expression of mesenchymal-specific genes, promoting the epithelial-to-mesenchymal transition, and cell migration.

### Deregulated Long Non-Coding RNAs in Parathyroid Carcinoma

Long non-coding RNAs (lncRNAs) are non-coding transcripts, longer than 200 nucleotides, acting as epigenetic regulators of gene expression, mainly in a tissue-specific fashion. A role of these molecules in regulating chromatin modifications and transcriptional and post-transcriptional gene expression has been demonstrated (53), as well as the fact that lncRNA deregulation is strongly associated with human tumorigenesis and cancer progression, through the activation of pro-oncogenic pathways and crosstalk with other RNA subtypes and epigenetic mechanisms (48). Alterations in lncRNA expression have been associated with endocrine diseases (54) and cancers (55, 56), including endocrine malignancies (57).

Up-regulation of two lncRNAs, [lncRNA PVT1 (47) and lncRNA BC200 (48)], and down-regulation of lncRNA GLIS2-AS1 (55), were significantly associated with PCs compared to healthy tissue and PAs. LncRNA BC200 has also been shown to discriminate PCs from aPAs, appearing to be a specific molecular signature of malignant tumors (48). Interestingly, lncRNA BC200 resulted significantly upregulated in the group of PCs with *CDC73* mutations compared to that of PCs with wild type parafibromin, in association with more aggressive clinical features, such as higher levels of PTH and calcium ion (48). Over-expression of lncRNA BC200 has been reported in a broad spectrum of tumor cells, being responsible for cell viability, invasion, and migration (58).

lncRNA PVT1 has been shown to promote cell proliferation of non-small cell lung cancer through the epigenetic regulation of the large tumor suppressor kinase 2 (LATS2) expression (59), by directly binding to EZH2, a transcriptional repression factor that has been shown to act as a pro-oncogenic factor in parathyroid carcinogenesis (9).

To date, there are no reports in cancer or other human diseases regarding lncRNA GLIS2-AS1, and the biological function of this lncRNA is still unknown. A possible parathyroid tissue-specific tumor suppressor activity could be considered and remains to be investigated.

### Circular RNA Expression Profile in Parathyroid Carcinoma

Circular RNAs (circRNAs) are a recently identified class of single-strand non-coding RNAs, consisting in a covalently closed loop generated by the 3’ and 5’ ends of the RNA molecule that have been joined together, which modulate miRNA activity by acting as miRNA sponges, regulate alternative splicing, and are suspected to have a role in human diseases and tumor progression. A microarray-based analysis of circRNA expression profile has been recently performed, for the first time, in human PAs, PCs and healthy parathyroids (60). Selected differentially expressed circRNAs and their corresponding mRNAs were validated by RT-qPCR, showing that 4 circRNAs (circRNA_0035563, circRNA_0017545, circRNA_0001687 and circRNA_0075005) and 4 mRNAs (MYC, FSCN1, ANXA2 and AKR1C3) were all significantly up-regulated in PCs than PAs, with circRNA_0075005 appearing the most promising diagnostic marker for distinguishing PC from PA. Expression of circ_0035563 and circ_0075005 was furtherly validated in formalin-fixed paraffin-embedded samples of PC and PA by fluorescence *in situ* hybridization, confirming both these molecules to be significantly higher in PC than in benign lesions. In addition, the study found circ_0035563 as significantly related to *CDC73* mutations and malignant recurrence, suggesting this parameter as a suitable marker of prognosis in PC patients. Results of this pivotal study open to the possibility of using circRNA expression as marker for the differential diagnosis of PC and PA; the effective clinical value of these molecules needs to be confirmed in further studies on larger sample size from multiple centers, to be analyzed in association with histopathological and clinical features of tumors, and to be functionally studied on PC cell lines.

## Gene Expression Profile and Proteomic Analysis in Parathyroid Carcinoma

Given the lack of specific PC biomarkers in clinical practice, two recent studies have investigated: 1) gene expression profile (61), and 2) global proteomic expression pattern (62), as possible molecular signatures to distinguish PC from PA.

The first study (61) compared the expression of 740 genes, known to be involved in main cancer progression processes [angiogenesis, extracellular matrix (ECM) remodeling, EMT, metastasis], between PAs, non-metastatic PCs, and metastatic PCs, identifying differentially expressed groups of genes able to discriminate PAs from PCs, and also differentiate non-metastatic PCs from metastatic PCs. Two specific signatures of 87 gene expressions (43 up-regulated and 44 down-regulated) and of 103 gene expressions (65 up-regulated and 38 down-regulated) characterized non-metastatic PCs and metastatic PCs, respectively, with respect to PAs. Among genes showing up-regulation in both metastatic and non-metastatic PCs *vs* PAs, there were genes encoding components of the ECM (*COL1A1, COL1A2, COL3A1, COL5A2, FN1, LUM, LOX, THBS1, THBS2, BGN*, and *SFRP2*) and genes encoding enzymes involved in proteolysis, cross-linking and assembly processes of proteins of the ECM (*FAP, LOX, SULF1* and *PXDN*), suggesting a key role of ECM deregulation in carcinogenesis, presumably altering cell proliferation (61). Metastatic PCs showed a significant up-regulation of *MMP9, SOX2, CD24, BMP7, ANGPTL4*, and *FGFR1* genes, and a down-regulation of *ERBB3, TBX1, RAB25*, and *FBP1* genes, compared to both PAs and non-metastatic PCs, suggesting a role of this gene expression signature in tumor progression and metastatic spread (61).

The second study (62) compared the global proteomic profile between a PC and a PA, co-existing in the same patient, by using two-dimensional differential gel electrophoresis coupled with mass spectrometry. The possibility to compare the two different types of tumors from the same patient allowed the identification of distinctive PC protein features without any external confounding influences, such as age, sex, germline background, lifestyle, and environmental factors. PC showed the specific over-expression of 23 proteins and the specific under-expression of 10 proteins, mainly belonging to specific biological processes, such as cell metabolism, cell signaling, and protein ubiquitination. In particular, the Ubiquitin C-terminal Hydrolase L1 (UCH-L1), a member of the deubiquitinase family involved in processing ubiquitin precursors and in regulating angiogenesis (63), and whose up-regulation has previously been demonstrated to enhance cell invasion/metastasis (64, 65), and increase multidrug resistance in breast cancer (66, 67), resulted highly over-expressed in PC, suggesting an oncogenic role of this protein in the malignant carcinogenesis of parathyroid tissue and, at the same time, indicating UCH-L1 as a possible distinctive immunohistochemical signature of PC with respect to benign parathyroid tumors. Other promising protein biomarkers, all over-expressed in PCs, were: Malate Dehydrogenase 1 (MDH1), an enzyme that catalyzes the NAD/NADH-dependent reversible oxidation of malate to oxaloacetate in many metabolic pathways and was showed to be up-regulated in cancer (68); Chloride Intracellular Channel Protein 1 (CLIC1), a protein involved in regulating cell proliferation and differentiation, and metastasis (69, 70), and whose up-regulation has previously been associated with human malignancies; and Superoxide Dismutase 2 (SOD2), which is a key player of the antioxidant system of the cell and whose increased expression appears correlated to the stage of tumor progression (71).

## Concluding Remarks

- Pre-operative diagnosis of PCs is complicated. There are no specific histological and molecular features helping to pre-operatively distinguish PAs, aPAs, and PCs.
- Loss-of-function mutations and/or loss of the *CDC73* tumor suppressor gene are the main genetic defects in PCs, both in the sporadic form and in the context of FIPH or HPT-JT syndrome. However, the absence of nuclear staining for parafibromin in tumor samples is not sufficient, alone, for a histological diagnosis of PCs.
- Hypermethylation of specific gene promoters has been specifically associated with PC, leading to gene silencing and presumably triggering parathyroid carcinogenesis. Theoretically, treatment with inhibitors of DNA methyltransferase would be expected to reduce the methylation density at hypermethylated promoters, restoring the normal expression of silenced genes. However, the anti-oncogenic beneficial of these molecules is controversial, since they exert a non-specific cytosine demethylation action that cannot be specifically directed on the target hypermethylated promoters. This may lead to a global DNA hypomethylation, increasing the risk of DNA damages.
- Deregulation of expression of specific miRNAs showed a promising capability to discriminate parathyroid tumors from healthy glands, as well as among different types of parathyroid tumors. MiRNA signatures specifically characterizing PCs have been identified.
- Down-regulation of miR-296-5p and miR-126-5p and up-regulation of miR-517c appear to be valuable molecular discriminators between PCs and PAs. These molecules might be, thus, valuable molecular targets for epigenetic therapy of PCs.
- Target inhibition of up-regulated miRNAs having an oncogenic activity (oncomiR) may be a valuable approach for epigenetic therapy of PC, which can be easily obtained by small molecule miRNA inhibitors, miRNA sponges, miRNA masks, or synthetic, modified antisense anti-miRNAs.
- The deregulated expression of specific lncRNAs (lncRNA PVT1, lncRNA GLIS2-SA1, and lncRNA BC200) also appears to characterize molecular signatures of human PCs, distinguishing between benign and malignant parathyroid tumors. However, the real contribution of lncRNAs to parathyroid carcinogenesis remains to be fully elucidated.
- The interplay between different epigenetic mechanisms, and their possible synergic action in promoting parathyroid carcinogenesis, should be studied as well.
- Epigenetic deregulation, rewiring the cell translational network, is thought to initiate PC tumorigenesis, by inducing a dedifferentiated tumor phenotype, characterized by an “embryonic-like” gene expression signature. Mutated oncogenes and tumor suppressor genes are suspected to contribute to parathyroid cancer development, presumably not only by inducing an increased cell proliferation, but also because of their role in deregulating the cell epigenome.
- Further investigations, to identify and/or confirm deregulated epigenetic factors specifically characterizing PCs, are needed, in association with functional *in vitro* studies, to understand their role in PC initiation, development and progression. The high-throughput “omic” technologies (genomics, transcriptomics, proteomics, metabolomics) will allow collective quantification and characterization of wide numbers of biological molecules, at different molecular levels, helping in deciphering the regulatory networks, whose deregulation underlies the development and progression of parathyroid cancer, and in identifying novel therapeutic targets.

## Author Contributions

FM wrote the manuscript and drew tables and figure. FG and GPa performed the review of published literature and contributed to drawing tables and figure. GPe, RS, and MB critically reviewed the manuscript. All authors contributed to the article and approved the submitted version.

## Conflict of Interest

The authors declare that the research was conducted in the absence of any commercial or financial relationships that could be construed as a potential conflict of interest.

## Publisher’s Note

All claims expressed in this article are solely those of the authors and do not necessarily represent those of their affiliated organizations, or those of the publisher, the editors and the reviewers. Any product that may be evaluated in this article, or claim that may be made by its manufacturer, is not guaranteed or endorsed by the publisher.
